# Correlation Between Radiological Features of Axillary Lymph Nodes with CD4 Count and Plasma Viral Load in Patients with HIV

**DOI:** 10.3390/tomography12010003

**Published:** 2025-12-25

**Authors:** Gulten Taskin, Muzaffer Elmali, Aydin Deveci, Irem Ceren Koc

**Affiliations:** 1 Department of Radiology, Faculty of Medicine, Ondokuz Mayıs University, 55270 Samsun, Turkey; 2 Department of Infectious Diseases and Clinical Microbiology, Faculty of Medicine, Ondokuz Mayıs University, 55270 Samsun, Turkey

**Keywords:** HIV infections, Axillary lymph nodes, computed tomography, CD4 lymphocyte count, viral load

## Abstract

**Objective:** Axillary lymph node changes are frequently observed in patients with HIV, yet their radiological characteristics and clinical significance remain underexplored. This study aimed to evaluate the association between axillary lymph node computed tomography (CT) features and clinical markers of immune function, including CD4 lymphocyte count and plasma viral load, in HIV-positive patients. **Materials and Methods:** In this retrospective study, 113 HIV-positive patients who underwent contrast-enhanced chest CT were included. Patients were stratified by CD4 count (<200, 200–500, >500 cells/μL) and plasma viral load (<100,000 or >100,000 copies/mL). Axillary lymph node parameters—including maximum and minimum diameters, cortical thickness, hilar width, and density (Hounsfield units, HU)—were measured on multiplanar reconstructed CT images. Group differences were assessed using the Kruskal–Wallis and Mann–Whitney U tests, and Spearman’s correlation was used to evaluate associations between imaging and laboratory findings. Receiver operating characteristic (ROC) curve analysis identified optimal density thresholds. **Results:** Lymph node diameters, cortical thickness, and hilar width did not significantly differ between CD4 groups. However, mean lymph node density was higher in patients with CD4 < 200 cells/μL (*p* = 0.024). A density threshold of 84.5 HU distinguished impaired from preserved immune function (sensitivity 61.1%, specificity 71.2%). Patients with viral load >100,000 copies/mL showed increased lymph node density, minimal diameter, and cortical thickness. **Conclusions:** Elevated axillary lymph node density correlates with immune suppression and high viral load, suggesting its potential as a non-invasive prognostic imaging biomarker in HIV infection.

## Simple Summary

In radiological practice, axillary lymph nodes in individuals living with HIV often display structural alterations, yet the relevance of these findings to immune status is not clearly defined. This study investigated whether CT-based characteristics of these lymph nodes reflect laboratory markers of immunosuppression, including CD4 count and viral load. Our results showed that lymph node density increases in patients with lower CD4 levels and higher viral load. These observations indicate that routine CT assessment may provide additional insight into immune function and help guide future research on imaging biomarkers in HIV infection.

## 1. Introduction

Human immunodeficiency virus (HIV) is a lymphotropic virus that primarily targets lymphoid tissues, with lymph nodes playing a central role in viral replication and disease pathogenesis [1]. Widespread lymphadenopathy is a common finding in HIV-infected individuals, most frequently involving the head and neck region and the axillary lymph nodes [2]. Superficial lymph node involvement is typically associated with reactive or persistent lymphadenopathy, whereas deep lymph nodes are more often affected by opportunistic infections or malignancies [3]. Progressive depletion of CD4+ lymphocytes within lymph nodes contributes to immune dysfunction and disease progression in HIV infection [4].

Clinically, HIV-related immunodeficiency is commonly categorized into early (CD4 > 500/μL), intermediate (CD4 200–500/μL), and advanced stages (CD4 < 200/μL), with opportunistic infections and malignancies predominantly occurring in advanced disease [3,5]. Plasma HIV viral load and CD4 lymphocyte count remain the principal laboratory biomarkers used to monitor immune status, assess disease progression, and guide clinical management in HIV-positive patients [6].

Although lymph node involvement in HIV-positive individuals has been extensively investigated using cytological, pathological, and nuclear medicine approaches [2,7,8,9,10,11,12], radiological studies focusing on lymph node characteristics remain limited [3,13]. Chest CT is frequently performed in HIV-positive patients due to common respiratory manifestations [14]; however, the potential value of CT-based lymph node features as imaging correlates of immune status has not been sufficiently explored. In particular, there are no dedicated radiological studies specifically evaluating axillary lymph node characteristics in relation to CD4 count and viral load. Therefore, the aim of this study was to investigate the association between CT-based radiological features of axillary lymph nodes and immunological markers, including CD4 lymphocyte count and plasma viral load, in patients with HIV.

### 1.1. Materials and Method

This retrospective, single-center study was conducted in accordance with the Declaration of Helsinki and the protocol was approved by the Institutional Ethics Committee (No. 2025/106). Due to the retrospective nature of the study, the requirement for informed consent was waived by the Institutional Ethics Committee, in accordance with local regulations.

### 1.2. Subjects

**Figure 1:**
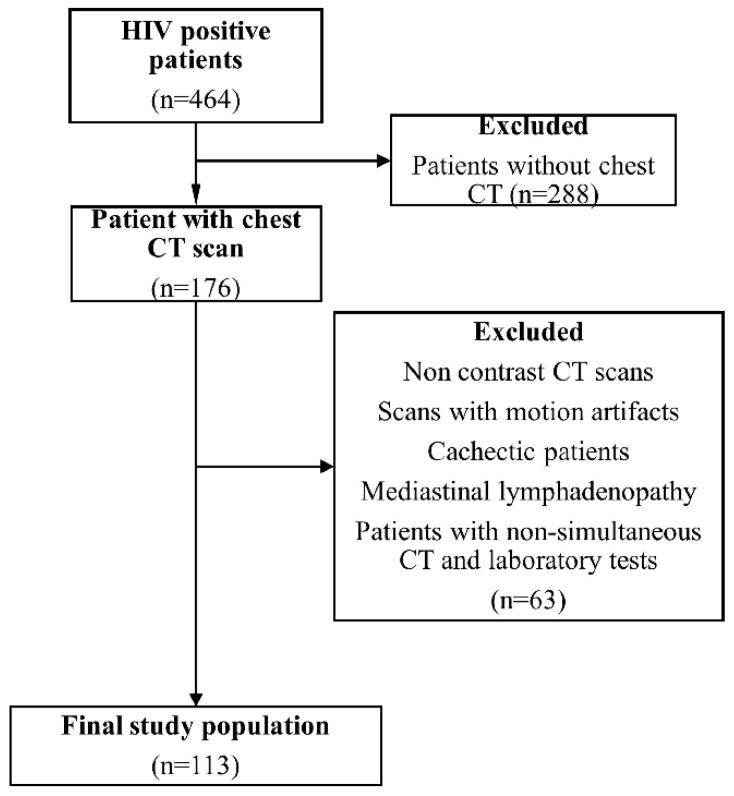
Flowchart of patient selection and exclusion criteria. Of 464 HIV-positive patients screened, 176 underwent contrast-enhanced chest CT, and 113 were included in the final analysis after exclusions.

Between 2014 and 2024, a total of 464 HIV-positive patients diagnosed in the Infectious Diseases and Clinical Microbiology Department were retrospectively screened from the institutional database. Among these, 176 patients who underwent chest CT were identified. Patients were excluded if CT images were non-contrast-enhanced, of insufficient quality (motion artifacts or non-measurable lymph nodes), if mediastinal lymphadenopathy was present due to increased risk of opportunistic infection or malignancy, or if laboratory data (CD4 count and viral load) were not available within 1–7 days of the CT examination. These laboratory measurements were obtained either before or after the CT examination within this time window, and no changes in antiretroviral therapy were recorded during this interval. After applying these criteria, 113 patients were included in the final analysis (Figure 1). All chest CT examinations included in this study were contrast-enhanced and performed for clinical indications.

Patients were classified into three groups based on their CD4 count: <200/μL, 200–500/μL, and >500/μL. Based on plasma HIV-RNA levels, the patients were further divided into two groups: low viral load (<100,000 copies/mL) and high viral load (>100,000 copies/mL).

### 1.3. CT Examination

All CT scans were performed using a 64-slice multidetector CT scanner (Discovery CT750 HD, GE Healthcare, Milwaukee, WI, USA) and a 160-slice helical CT scanner (Aquilion Prime SP, Canon Medical Systems, Otawara, Japan) following a standard thoracic CT protocol that covered the area from the lung apex to the diaphragm. For the first scanner, the acquisition parameters were as follows: Kv: 100–120, mA: 150–350, gantry rotation time: 0.4 s, slice thickness: 2.5 mm, slice interval, 0.625 mm. For the second scanner, the acquisition parameters were: Kv: 120, mA: 250; gantry rotation time: 0.4 s, slice thickness, 1 mm; and slice interval, 0.625 mm. All contrast-enhanced chest CT examinations were performed using a standard clinical protocol with intravenous administration of a non-ionic iodinated contrast agent at an injection rate of 2 mL/s. A fixed contrast volume ranging between 70 and 100 mL was used, depending on patient body habitus and clinical indication. Images were acquired during the contrast-enhanced phase according to routine institutional practice.

### 1.4. CT Measurement

Chest CT examinations were performed by a radiologist with 15 years of experience in thoracic imaging. All evaluations were performed using Horos software (version 3.3.6, Horos Project, Geneva, Switzerland). The maximum and minimum diameters, cortical thickness, hilar width, and cortical density of the axillary lymph nodes were measured on three-dimensional (3D) multiplanar reformatted (MPR) images. Axillary lymph nodes were assessed at three levels (levels I–III), and measurements were obtained from the largest lymph node on either the right or left side, using axial, sagittal, and coronal planes, with measurements performed perpendicular to the long axis of the lymph node on the slice demonstrating the thickest cortical portion. For cortical density measurements, a region of interest (ROI) was manually placed within the thickest or most asymmetrically thickened portion of the lymph node cortex on the selected slice. Care was taken to avoid inclusion of the fatty hilum, adjacent perinodal adipose tissue, and visible vascular structures in order to minimize partial volume effects. Two to three separate measurements were obtained for each lymph node, and the mean value was used for statistical analysis.

The largest axillary lymph node was selected to ensure measurement standardization and reproducibility across patients, allowing consistent comparison of radiological parameters.

To evaluate intraobserver reliability, measurements were repeated by the same radiologist after a two-week interval to minimize recall bias. Intraobserver agreement was assessed using the intraclass correlation coefficient (ICC) based on repeated measurements.

### 1.5. Statistical Analysis

All statistical analyses were performed using SPSS Statistics (version 26.0, IBM Corp., Armonk, NY, USA). For the comparison of maximum and minimum lymph node diameter, maximum cortex thickness, hilum width, and cortex density across the three CD4 groups, the Kruskal–Wallis test was applied due to non-normal distribution, as determined by the Shapiro–Wilk test. Post hoc tests were conducted to determine which group differences were statistically significant in the parameters where significant differences were found. The Mann–Whitney U test was used for comparisons involving viral load, as the normal distribution was not met. The relationship between CD4 count, viral load, and radiological parameters was assessed using Spearman’s correlation test, with the significance level set at *p* < 0.05. Additionally, to evaluate the potential clinical utility of cortex density measured on contrast-enhanced scans, Receiver Operating Characteristic (ROC) curve analysis was performed for CD4 counts with normal (>500/μL) and low (<500/μL) values to determine the threshold value of density. The point with the best combination of sensitivity and specificity was considered the optimal cutoff value. The area under the ROC curve (AUC) was used to assess the predictive validity of cortical density.

## 2. Results

### 2.1. Demographic Findings

The final study population comprised 113 patients (101 men and 12 women). The mean patient age was 47.99 ± 12.61 years (range: 22–79 years). Among the participants, 60 had a CD4 count < 200, 35 had a CD4 count between 200 and 500, and 18 had a CD4 count > 500. The chi-square test revealed no significant sex differences among the three groups (*p* = 0.119). Of the participants, 26 had a viral load > 100,000 copies/mL, while 87 had a viral load < 100,000 copies/mL. When the participants were categorized into two groups based on viral load, no statistically significant sex differences were found between the two groups (*p* = 0.630).

### 2.2. Intraobserver Reliability Findings

In our study, intraobserver reliability for measurements of maximum diameter, minimum diameter, density, hilum width, and cortical thickness was assessed using the intraclass correlation coefficient (ICC). The ICC values were calculated as follows: 0.92 (CI: 0.88–0.95), 0.85 (CI: 0.81–0.90), 0.80 (CI: 0.75–0.86), 0.78 (CI: 0.72–0.84), and 0.90 (CI: 0.86–0.94), respectively. All measurements had *p*-values of <0.001, indicating statistical significance. These results demonstrate a high degree of intraobserver reliability for the evaluated parameters.

### 2.3. Radiological Findings by CD4 Count

**Figure 2:**
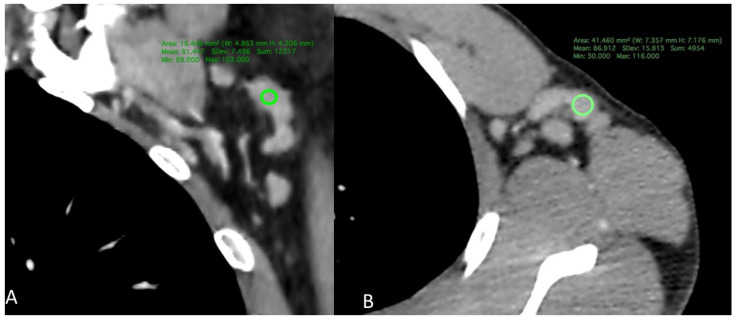
Contrast-enhanced chest CT examples of axillary lymph node cortical density measurements in HIV-positive patients. (**A**) CD4 count > 500 cells/μL; (**B**) CD4 count < 200 cells/μL.

**Figure 3:**
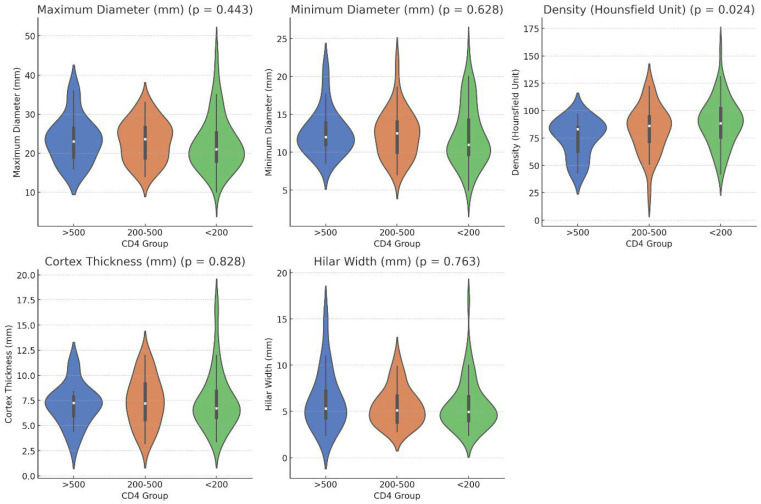
Violin plots of axillary lymph node maximum diameter, minimum diameter, cortical density (HU), cortical thickness, and hilar width across CD4 groups (<200, 200–500, and >500 cells/μL). *p*-values are from Kruskal–Wallis tests.

**Figure 4:**
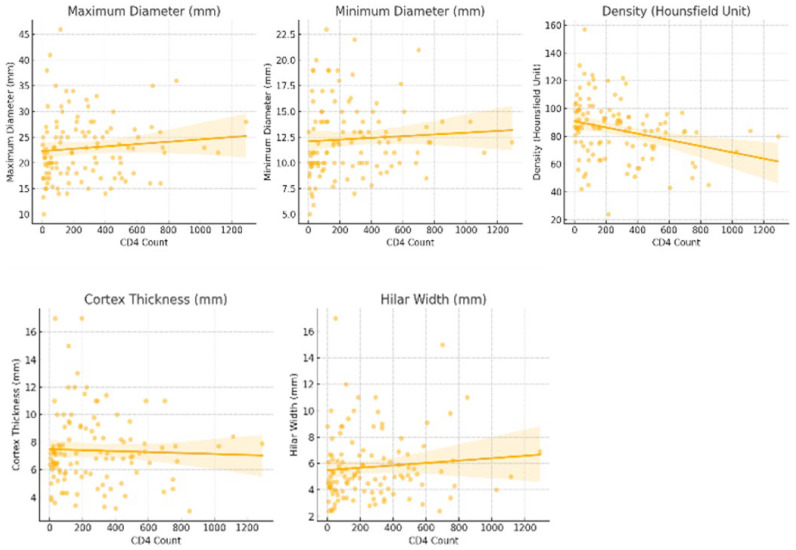
Scatter plots of CD4 count versus axillary lymph node maximum diameter, minimum diameter, cortical density, cortical thickness, and hilar width. Lines indicate linear regression fits.

When considering CD4 counts, no statistically significant differences were observed in the maximum diameter, minimum diameter, cortex thickness, or hilum width according to the Kruskal–Wallis test (*p* = 0.443, 0.628, 0.828, and 0.763, respectively). However, regarding density, the group with CD4 < 200 had a density of 89.12 ± 21.88 HU, the CD4 200–500 group had a density of 82.89 ± 21.22 HU, and the CD4 > 500 group had a density of 75.17 ± 16.97 HU (Figure 2). The Kruskal–Wallis test revealed a statistically significant difference between the groups (*p* = 0.024). Post hoc tests indicated that the CD4 > 500 group had a significantly lower density than the CD4 < 200 group (adjusted *p* = 0.022). The comparison between the CD4 > 500 and CD4 < 200 groups yielded an adjusted *p*-value of 0.022, whereas the comparison between the CD4 200–500 and CD4 < 200 groups resulted in an adjusted *p*-value of 0.637 (Table 1, Figure 3 and Figure 4).

### 2.4. CD4 Count and Density as a Predictor

**Figure 5:**
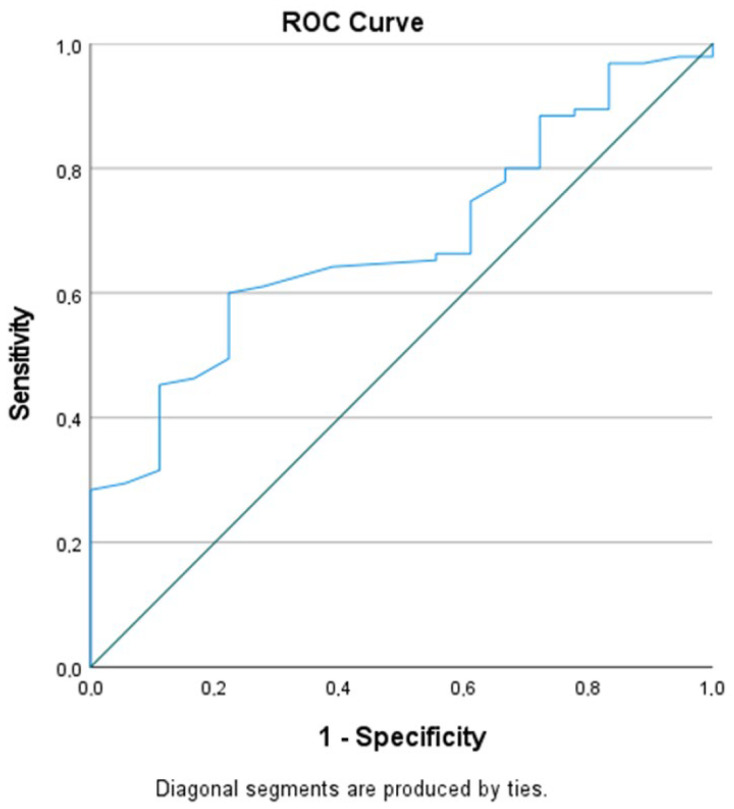
Receiver operating characteristic (ROC) curve of axillary lymph node cortical density for discrimination between preserved (CD4 > 500 cells/μL) and impaired (CD4 < 500 cells/μL) immune function on contrast-enhanced chest CT (AUC = 0.681). The solid line represents the ROC curve, while the diagonal line represents the reference line indicating no discrimination (AUC = 0.5).

Given that only density showed significant variability across the CD4 groups, its potential as a distinguishing predictor of CD4 status was further investigated. Receiver operating characteristic (ROC) analysis demonstrated that a density threshold of 84.5 HU could differentiate individuals with normal CD4 counts (CD4 > 500) from those with abnormal counts (CD4 < 500) with a sensitivity of 61.1% and specificity of 71.2% (AUC: 0.681 [95% Confidence Interval (CI): 0.565–0.797], *p* = 0.015) (Figure 5).

### 2.5. Radiological Findings by Viral Load

**Figure 6:**
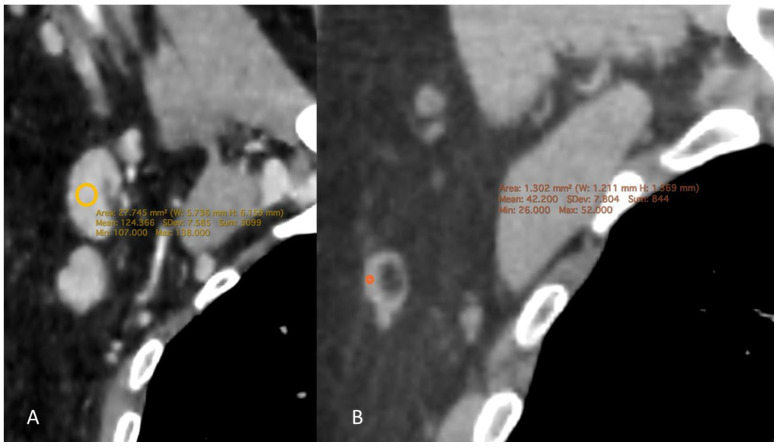
Contrast-enhanced chest CT coronal multiplanar reconstruction images demonstrating axillary lymph node cortical density in HIV-positive patients. (**A**) High plasma viral load; (**B**) Low plasma viral load.

In patients with low viral load, the average minimum lymph node diameter was 11.91 ± 3.34 mm, while in patients with high viral load, the minimum lymph node diameter was 13.73 ± 4.09 mm, showing a statistically significant increase in the high viral load group (*p* = 0.046). The average cortex thickness was 7.09 ± 2.43 mm in the low viral load group, compared to 8.71 ± 3.00 mm in the high viral load group, with a statistically significant increase in the high viral load group (*p* = 0.008). The average cortex density was 81.53 ± 20.23 HU in the low viral load group, whereas in the high viral load group, it was 97.04 ± 21.47 HU, which was also statistically significantly higher (*p* = 0.002) (Figure 6).

**Figure 7:**
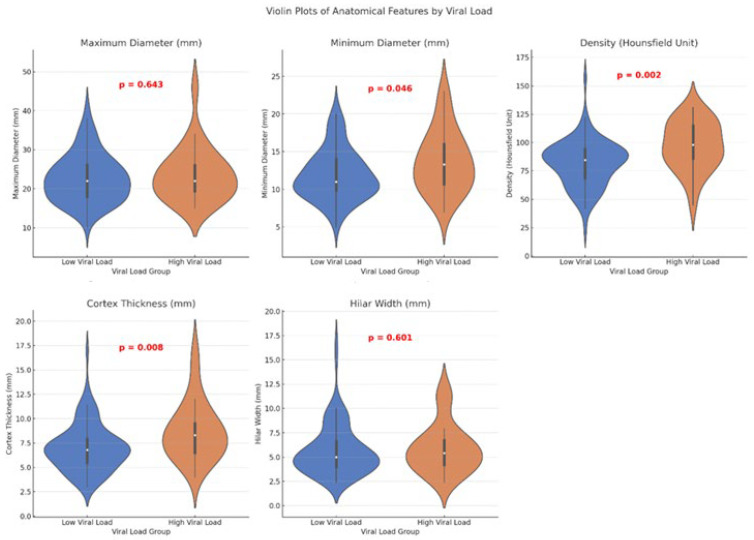
Violin plots comparing axillary lymph node maximum diameter, minimum diameter, cortical density, cortical thickness, and hilar width between low and high plasma viral load groups. *p*-values are from Mann–Whitney U tests.

**Figure 8:**
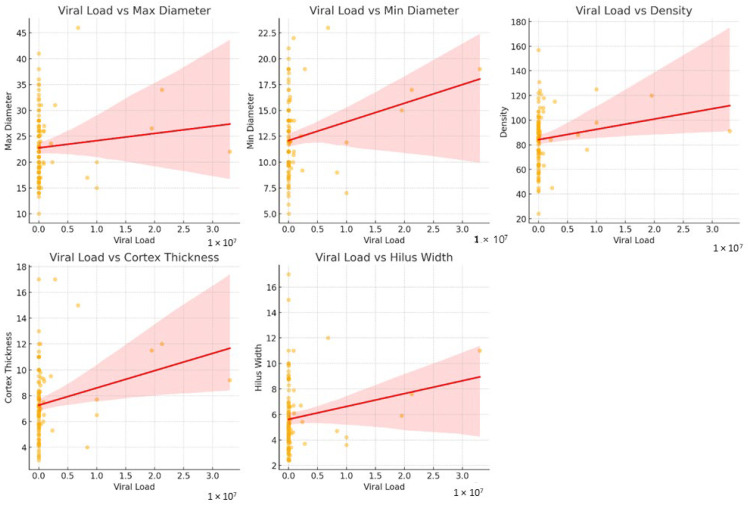
Scatter plots showing the associations between plasma viral load and axillary lymph node maximum diameter, minimum diameter, cortical density, cortical thickness, and hilar width. Lines indicate linear regression fits with 95% confidence intervals.

No statistically significant difference was found in hilum width between the low (5.69 ± 2.61 mm) and high viral load groups (5.86 ± 2.51 mm). Similarly, the maximum diameter was 22.68 ± 6.18 mm in the low viral load group and 23.58 ± 6.88 mm in the high viral load group, with no significant difference (Table 2, Figure 7 and Figure 8).

**Figure 9:**
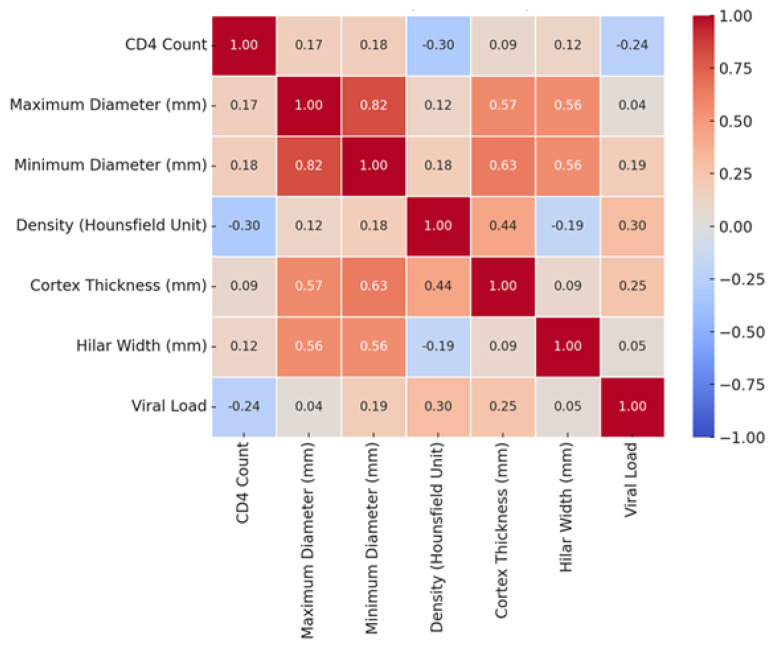
Correlation heatmap illustrating the relationships between CD4 count, plasma viral load, and axillary lymph node morphometric parameters measured on contrast-enhanced chest CT.

Correlation of CD4 Count, Viral Load, and Radiological Parameters:

Spearman’s correlation tests revealed a statistically significant weak negative correlation between viral load and CD4 count (rho = −0.257, *p* = 0.006). CD4 count showed a moderate negative correlation with cortical density (rho = −0.300, *p* = 0.001). Viral load was positively correlated with cortical density (rho = 0.310, *p* = 0.001) and cortical thickness (rho = 0.300, *p* = 0.001). No statistically significant correlations were found between viral load and maximum diameter or hilar width, or between CD4 count and lymph node size parameters (*p* > 0.05) (Figure 9).

## 3. Discussion

Lymph nodes, particularly in the axillary region, along with other lymphoid tissues are the primary sites of HIV infection. Superficial lymph node involvement is more commonly associated with reactive or persistent lymphadenopathy, whereas deep lymph node involvement tends to be secondary to opportunistic infections or malignancies [15,16]. The CD4 lymphocyte count serves as a crucial biomarker for assessing immune function in HIV-positive individuals, whereas plasma HIV viral load reflects the level of viral activity [17].

Although HIV-positive individuals may not exhibit clinical signs of opportunistic infections or malignancies, chest CT is frequently used in clinical practice owing to the common occurrence of respiratory symptoms. However, to date, there has been a paucity of studies identifying radiological parameters, particularly those derived from chest CT, that predict the clinical status of HIV-positive patients with axillary lymph node involvement.

Our study underscores the importance of lymph node cortical density on chest CT as a significant radiological marker reflecting both immune status and clinical progression in HIV-positive individuals. We observed a statistically significant increase in lymph node cortical density in patients with a CD4 lymphocyte count <200/μL (*p* = 0.024). Similarly, individuals with a high viral load (>100,000 copies/mL) also exhibited a statistically significant increase in lymph node density (*p* = 0.002). These findings suggest that elevated lymph node density in superficial lymph nodes, such as those in the axillary region, correlates with immune system deterioration and heightened viral activity in HIV-positive patients. Therefore, we propose that increased lymph node density may serve as a valuable radiological predictor of adverse clinical outcomes in this population.

Structural changes in the lymph nodes of HIV-positive individuals are commonly associated with persistent immune activation, ongoing tissue inflammation, and resultant fibrosis [18]. HIV infection manifests in lymph nodes at three histological stages: acute, subacute, and chronic, reflecting the clinical status of the patient. In the acute phase, enlarged lymphoid follicles with prominent germinal centers were observed. The subacute phase is characterized by progressive damage to germinal centers, lymphocyte reduction, and obliteration of follicular structures. In the chronic phase, lymphoid follicles exhibit hyalinization, small germinal centers, severe lymphocyte depletion or exhaustion, increased plasma cell numbers, and excessive vascular proliferation [19]. Based on these findings, we hypothesized that the increase in lymph node density observed in patients with low CD4 counts is associated with fibrosis resulting from lymph node destruction and hyalinization, as well as excessive vascular proliferation. Increased vascularity, particularly in contrast-enhanced imaging, leads to greater contrast uptake in the lymph nodes, thereby contributing to higher density measurements.

Moreover, a high viral load reflects active viral replication and has been shown to correlate negatively with CD4 count. This relationship may further explain the observed changes in lymph node density, as both viral load and CD4 count influence the structural integrity and vascular characteristics of the nodes.

Previous studies have indicated that even in individuals receiving antiretroviral therapy (ART), immune parameters such as CD4 counts, immune activation, and suppressed viremia may partially normalize, whereas structural changes in the lymph nodes persist [20,21]. As structural alterations in the lymph nodes continue independent of ART, we did not investigate the impact of treatment status on radiological parameters in the current study.

In routine radiological practice, various parameters are used to differentiate between pathological and reactive lymph nodes. These include changes in the maximum and minimum diameters, shape (oval or round), asymmetric versus symmetric cortical thickening, partial or complete loss of the fatty hilum, and distortion or irregularity of the outer contours of lymph nodes. Previous studies evaluating cross-sectional imaging methods (CT and MRI) for assessing metastatic lymph nodes, particularly in patients with breast cancer, have demonstrated that high-resolution or thin-slice imaging combined with multiplanar reconstruction is effective in distinguishing between malignant and benign nodes [22,23]. Given the relevance of these parameters, we hypothesized that they might also be valuable for evaluating immune status and clinical prognosis in HIV-positive individuals, a notion we examined in this study.

However, our results revealed no significant differences in the maximum or minimum diameter, cortical thickness, or hilum width between groups stratified by CD4 lymphocyte counts. Consequently, these parameters do not appear to be reliable radiological markers of immune system status in HIV-positive individuals. We suggest that the different mechanisms underlying structural changes in the lymph nodes due to the lymphotropic nature of HIV may account for the absence of significant findings. In contrast to malignancies in which metastasizing cells induce an increase in nuclear size, cortical thickening, and hilum narrowing, resulting in overall lymph node enlargement, HIV disrupts the follicular architecture, causes hyalinized tissue formation, and depletes CD4 +cells. Thus, the expected radiological changes in lymph node size, cortical thickness, and hilum width may not occur in HIV-positive individuals unless opportunistic infections or malignancies develop.

In our study, the only significant radiological parameter that varied according to CD4 count was lymph node cortical density. We found that lymph node density was increased in patients with low CD4 counts and decreased in those with high CD4 counts. Based on these findings, we explored the potential of lymph node density as a parameter to distinguish between individuals with normal (CD4 > 500/μL) and abnormal (CD4 < 500/μL) immune functions. We identified a density threshold of 84.5 HU that effectively differentiated these two groups, with a sensitivity of 61% and specificity of 71%. The area under the curve (AUC) for this threshold was 0.681, with a 95% confidence interval (CI) of 0.565–0.797, and a statistically significant *p*-value of 0.015. To our knowledge, this is the first study to propose a CT-based density threshold for axillary lymph nodes in relation to CD4 count. However, given the moderate discriminative performance (AUC = 0.681), cortical density should be considered a supportive imaging parameter rather than a stand-alone marker of immune status in HIV-positive individuals.

When evaluating radiological parameters based on viral load, which reflects HIV viral activity, we found that in addition to cortical density, classic parameters such as the minimum diameter and cortical thickness of lymph nodes were significantly increased in patients with high viral loads. These findings suggest that such parameters may be valuable in assessing the impact of viral activity on lymph node morphology.

Our study has several limitations. First, its retrospective nature introduces potential bias. In this study, measurements were obtained from the largest axillary lymph node to improve reproducibility and reduce inter-measurement variability; however, this approach may not fully reflect regional nodal heterogeneity. Additionally, the heterogeneity of the cohort and the lack of pathological results for most lymph nodes are notable limitations. In addition, variability in antiretroviral treatment status, duration of HIV infection, and the presence of concomitant inflammatory or metabolic conditions may have acted as potential confounding factors and could not be fully controlled for in this retrospective design. Consequently, we could not definitively exclude opportunistic infections or malignancies that might influence lymph node density nor could we assess their impact. However, as noted in the previous literature [16], we excluded patients with mediastinal lymphadenopathy, which likely minimized the inclusion of individuals with a high likelihood of opportunistic infections or malignancies. Furthermore, opportunistic infections such as tuberculosis, which frequently affect HIV-positive individuals with low CD4 counts, can result in necrosis and reduced lymph node density [3]. Although different scanners and acquisition parameters were used, all examinations were performed using standardized clinical protocols, and potential variability in attenuation values was considered a technical limitation of the study. Although the unequal distribution of patients across viral load groups limited our ability to define a precise threshold value, this did not significantly affect the statistical validity of our results regarding the radiological parameters. In addition, the interpretation of the observed correlations should be approached with caution due to data distribution and clustering. Therefore, these correlations should be considered exploratory rather than definitive.

Moreover, while some of our cohort received regular ART and others had inconsistent treatments, the primary focus of our study was on morphological changes. Previous studies have demonstrated that structural changes in lymph nodes persist even in patients receiving ART [21], suggesting that inconsistent treatment would not have substantially impacted our results.

This study is the first to evaluate axillary lymph nodes in HIV-positive individuals using radiological methods, emphasizing the need for future prospective studies with larger cohorts, equal distributions of demographic and laboratory values, and supporting pathological results. These studies should include lymph nodes from various anatomical regions to validate and expand our findings.

## 4. Conclusions

In HIV-positive patients, axillary lymph node cortical density on contrast-enhanced chest CT was associated with both CD4 lymphocyte count and plasma viral load, with higher density observed in individuals with greater immune suppression. Although its discriminative performance was moderate (AUC = 0.681), cortical density may serve as a supportive imaging parameter rather than a stand-alone marker of immune status. Clinically, routine assessment of this parameter may provide non-invasive, complementary information to laboratory markers in the radiological evaluation of HIV-positive patients.

## Figures and Tables

**Table 1:** Correlation Between CD4 Count and Radiological Parameters.

Parameters *	CD4 < 200 (n = 60)	CD4 > 200; CD4 < 500 (n = 35)	CD4 > 500 (n = 18)	*p* Value **
Maximum Diameter (mm)	22.55 ± 7.09 21 (7.8)	23.14 ± 5.20 23.60 (8.2)	23.48 ± 5.84 23 (8.9)	0.443
Minimum Diameter (mm)	12.18 ± 3.98 11 (5.1)	12.40 ±3.19 2.5 (3.19)	12.59 ± 3.02 12 (3.3)	0.628
Density (Hounsfield Unit)	89.12 ± 21.88 88.5 (27)	82.89 ± 21.22 86 (24)	75.17 ± 16.97 83 (26)	0.024 ***
Cortex Thickness (mm)	7.48 ± 2.94 6.7 (3.1)	7.43 ± 2.45 7.2 (3.8)	7.05 ± 2.03 7.25 (2.2)	0.828
Hilar Width (mm)	5.66 ± 2.66 4.95 (2.6)	5.57 ± 2.09 5.1 (2.9)	6.26 ± 3.20 5.3 (3.5)	0.763

* The parameters are presented as median (IQR) and mean standard deviation for each group. ** All comparisons were conducted using the Kruskal–Wallis test. *** Post hoc comparisons, following a significant Kruskal–Wallis test result, revealed that the group with CD4 counts >500 exhibited significantly lower density compared to the group with CD4 counts <200 (adjusted *p* = 0.022).

**Table 2:** Comparison Between Viral Load and Radiological Parameters.

Parameters	Low Viral Load (<100.000 Copies/mL) (n = 88)	High Viral Load (>100.000 Copies/mL) (n = 25)	*p* Value *
Maximum Diameter (mm)	22.68 ± 6.18 22 (8.4)	23.58 ± 6.88 22 (7.1)	0.643
Minimum Diameter (mm)	11.91 ± 3.34 11 (4.3)	13.73 ± 4.09 13.30 (5.8)	0.046
Density (Hounsfield Unit)	81.53 ± 20.23 84.50 (27)	97.04 ± 21.47 98 (32)	0.002
Cortex Thickness (mm)	7.09 ± 2.43 6.8 (2.5)	8.30 (3.2)	0.008
Hilar Width (mm)	5.69 ± 2.61 5 (2.7)	5.86 ± 2.51 5.4 (2.7)	0.601

* The Mann–Whitney U test was used for all comparisons.

## Data Availability

The raw data supporting the conclusions of this article will be made available by the authors on request.
